# Antibacterial Activity of Free Fatty Acids from Hydrolyzed Virgin Coconut Oil Using Lipase from* Candida rugosa*

**DOI:** 10.1155/2017/7170162

**Published:** 2017-11-13

**Authors:** Van Thi Ai Nguyen, Truong Dang Le, Hoa Ngoc Phan, Lam Bich Tran

**Affiliations:** ^1^Institute of Biotechnology and Food Technology, Industrial University of Ho Chi Minh City, Ho Chi Minh City, Vietnam; ^2^Department of Food Technology, Faculty of Chemical Engineering, Ho Chi Minh City University of Technology, Ho Chi Minh City, Vietnam

## Abstract

Free fatty acids (FFAs) were obtained from hydrolyzed virgin coconut oil (VCO) by* Candida rugosa* lipase (CRL). Four factors' influence on hydrolysis degree (HD) was examined. The best hydrolysis conditions in order to get the highest HD value were determined at VCO to buffer ratio 1 : 5 (w/w), CRL concentration 1.5% (w/w oil), pH 7, and temperature 40°C. After 16 hours' reaction, the HD value achieved 79.64%. FFAs and residual hydrolyzed virgin coconut oil (HVCO) were isolated from the hydrolysis products. They were tested for their antibacterial activity against Gram-negative and Gram-positive bacteria, which can be found in contaminated food and cause food poisoning. FFAs showed their inhibition against* Bacillus subtilis* (ATCC 11774),* Escherichia coli* (ATCC 25922),* Salmonella enteritidis* (ATCC 13076), and* Staphylococcus aureus* (ATCC 25923) at minimum inhibitory concentration (MIC) of 50%, 60%, 20%, and 40%, respectively. However, VCO and HVCO did not show their antibacterial activity against these tested bacteria.

## 1. Introduction

VCO is extracted from fresh kernel by using either cold press or centrifuge process. It does not go through refined, bleached, and deodorized process (RBD). Therefore, its physical properties as flavor, color, and so forth are less changed than RBD oil. VCO has many advantages in skin care, promotes the growth of hair, and enhances the beauty. Antioxidant activity and phenolic compounds in VCO also was conducted by some studies; it was suggested that the consumption of food containing phenolic compound will have a positive contribution in health [1].

Aside from benefits above, VCO is also good in health promotion and prevents some diseases because of the presence of FFAs in VCO. FFAs in VCO are rich in medium chain fatty acids (MCFAs) in which lauric acid takes the highest percentage about 46–48%. MCFAs in VCO are easily digested and absorbed, but fat is harder because it contains long chain fatty acids which need going through circulatory system before absorbing, so VCO can be used to replace cooking oil in daily meal to improve digestion. Moreover, MCFAs are also good for obese people because they increase energy expenditure more than usual. And MCFAs are directly absorbed from the intestine and burned in the liver; this makes them have a feeling which is always early satiety, and weight is decreased [2]. And also absorbing and burning directly in liver make MCFAs not take part in biosynthesis and transport of cholesterol. Thus, MCFAs in VCO have cardioprotective ability [3]. MCFAs also showed antifungal activity; Shino and coworkers (2016) exhibited a comparison of antimicrobial activity of chlorhexidine, coconut oil, probiotics, and ketoconazole on* Candida albicans* isolated in children with early childhood caries [4]. Parfene and coworkers (2013) gave a result about antifungal activity against* Yarrowia lipolytica* of MCFAs from crude coconut oil [5]. MCFAs have effective ability to inhibit some species of virus by breaking their membranes [3]. And antibacterial activity of MCFAs was also conducted by some previous studies; Kim and Rhee (2016) presented that MCFAs were antibacterial agents against* Escherichia coli* [6]. Shilling and coworkers (2013) also studied antimicrobial effect of VCO and MCFAs against* Clostridium difficile* [7].

MCFAs are antibacterial agents; this was demonstrated by previous studies, but MCFAs used in their studies were in form of pure chemical. Therefore, the aim of this study was to use FFAs extracted from hydrolyzed VCO and evaluate their antibacterial against* Bacillus subtilis* (ATCC 11774),* Escherichia coli* (ATCC 25922),* Salmonella enteritidis* (ATCC 13076), and* Staphylococcus aureus* (ATCC 25923), which can be found in food and cause food poisoning. At the same time, the resistance of VCO and HVCO against these tested bacteria was also evaluated.

## 2. Materials and Methods

### 2.1. Materials

VCO was sponsored by Luong Quoi Coconut Co., Ltd. (Ben Tre Province, Vietnam).* Candida Rugosa* lipase (CRL) (Type VII, ≥700 unit/mg solid) was purchased from Sigma-Aldrich Co. (Canada). Chemicals used in this study were KOH, n-hexane, and iso-octane and all other chemicals from Merck (Germany) and China were analyzed with purification more than 95%. Mediums used in antibacterial test were Nutrient Broth (NB) (Italy), Mueller Hinton Agar (MHA) from HiMedia Laboratories Pvt. Ltd (India), and Mueller Hinton Broth (MHB) from Titan Biotech Ltd (India). And four types of bacteria used in this study were* Bacillus subtilis* (ATCC 11774),* Escherichia coli* (ATCC 25922),* Salmonella enteritidis* (ATCC 13076), and* Staphylococcus aureus* (ATCC 25923) provided by Microbiologics Co. (USA).

Devices used in this study were high speed homogenizer (IKA T25 digital ULTRA-TURRAX, Germany), overhead stirrer (OS20, USA), orbital shaker incubator (LM-2575RD) from Yihder Technology Co. (Taiwan), evaporator (IKA RV digital V) from Germany, and GC-FID SHIMADZU 2010 Plus (Japan).

### 2.2. Hydrolysis of VCO

VCO dissolved in iso-octane (VCO to solvent ratio 1 : 1 (w/w)) and phosphate buffer solution to adjust pH condition was placed in a 250 mL Erlenmeyer flask [8]. Emulsification of the mixture was carried out by using a stirrer at speed of 10000 rpm in 15 minutes; then the appropriate amount of lipase was added and dissolved by stirring at speed of 350 rpm in 5 minutes. The reaction was conducted in orbital shaker incubator at speed 150 rpm for 2 hours. To stop the reaction, add 1 ml ethanol 99.5% into the erlenmeyer flask.

The hydrolysis degree (HD) was calculated as the following formula [9]:

(1) $$\begin{array}{c} HD=\frac{V_{KOH}*M_{KOH}*M_{FFAs}}{10*m}\left(\;\%\;\right), \end{array}$$

where *V*_KOH_ is the volume of potassium hydroxide (KOH) titrated (mL), *M*_KOH_ is the molarity of KOH solution (mol/L), *M*_FFAs_ is the average molecular weight of free fatty acids, and *m* is mass of VCO (g).

### 2.3. Obtaining FFAs

The process was carried out according to the method of Shimada and coworkers (1998) [10]. The excess of 0.5 N KOH was added to the hydrolyzed mixture to neutralize the released FFAs. Separatory funnel was used to extract FFAs and HVCO (tri-, di-, and monoglyceride). The upper phase containing HVCO dissolved in n-hexane was purified by using rotary evaporator. The lower phase containing FFAs was acidified with 4 N HCl solution and then was extracted by n-hexane. FFAs and HVCO were analyzed composition by Gas Chromatography with Flame Ionization Detector (GC-FID).

### 2.4. Antibacterial Activity Test

The test was conducted following disk diffusion method. Firstly, bacterial inoculum suspension was diluted in saline and was adjusted to get the final inoculum to 1.5 × 10^6^ CFU/mL [11]. Mix the volume of 1 mL bacterial inoculum to 15 mL of MHA and wait until the media solidified [12]. Paper discs with diameter of 6 mm containing different solutions such as gentamicin, VCO, HVCO, and FFAs were placed in agar surface and then were incubated for 24 hours at 37°C. The antibacterial activity was determined by measuring the diameter of inhibition zone.

### 2.5. Determining the Minimum Inhibitory Concentration (MIC)

MIC was conducted following the broth macrodilution method. Bacterial inoculum suspension was diluted directly in MHB and was adjusted to get final inoculum to 5 × 10^5^ CFU/mL. The antibacterial agent (FFAs) was prepared by diluting FFA directly in MHB to get dilution series 100%, 90%,…, 10%. 1 mL of bacterial inoculum was added to each tube containing 1 mL of dilution series of FFAs (positive control tube only has MHB) and mixed the mixture. The tubes were incubated at 35°C from 16 to 20 hours before recording. The MIC is the lowest concentration of FFAs that completely inhibits growth of the bacteria in the tube. It was compared with positive control tube when determining growth end points. The results of this test are valid if growth-control well is definitely turbid or diameter of colonies ≤ 2 mm [13].

All the experiments were carried out in triplicate and the data collected were statistically analyzed by using R software.

## 3. Results and Discussion

### 3.1. Effect of VCO to Buffer Ratio

**Figure 1:**
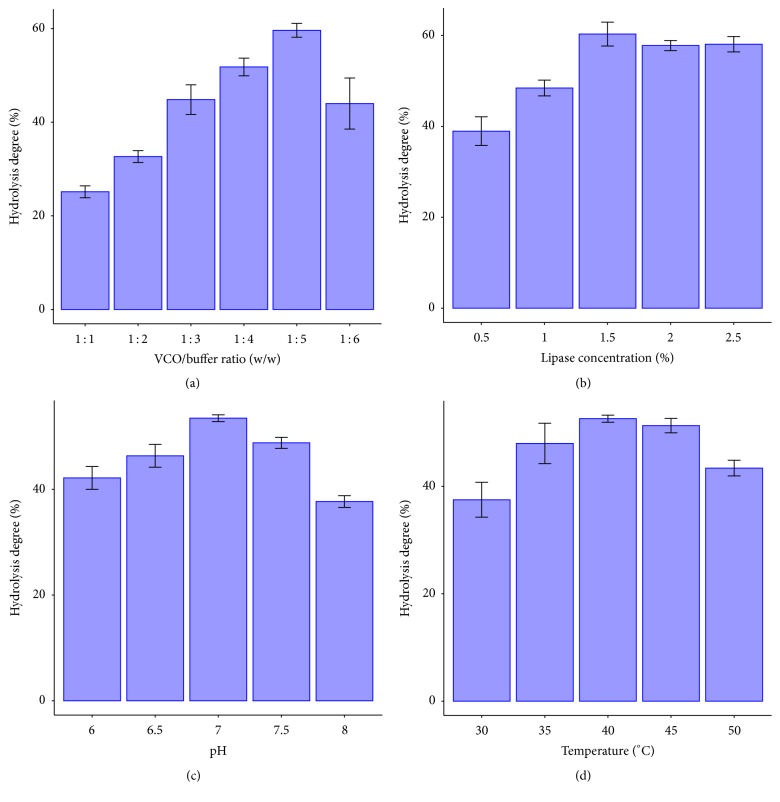
The rate of VCO to buffer (a), CRL concentration (b), pH (c), and temperature (d) effect on hydrolysis degree of VCO by CRL.

Lipase catalyzes hydrolysis reaction at interfacial area of emulsion. There are more bonds between water and oil when amount of buffer increases, so lipase has more substrates to be catalyzed leading to increase of hydrolysis degree. However, the excess of water amount will decrease hydrolysis degree because of substrate competition by binding to lipase [8]. In Figure 1(a) the VCO to buffer ratio of 1 : 5 (w/w) showed the highest hydrolysis degree. Therefore, the next experiment will be carried out at this ratio.

### 3.2. Effect of CRL Concentration

CRL concentration has a significant effect on hydrolysis degree. Increasing lipase concentration will get a high hydrolysis degree, but when the interface of emulsion is saturated with lipase concentration, adding more lipase will not get a higher hydrolysis degree [14]. From Figure 1(b) the hydrolysis degree cannot increase at CRL concentration of 1.5% plus anymore. As a result, the CRL concentration of 1.5% was chosen to carry out next experiments.

### 3.3. Effect of pH

Each lipase has an optimum pH condition. Changed pH range affects the ionization of substrate, free lipase, or lipase-substrate complex. Either high pH or low pH will make lipase denatured; substrate is broken down leading to a decrease of hydrolysis degree [15].

According to Figure 1(c), pH 7.0 was the best condition for hydrolysis reaction. And this result also was equivalent to some studies as Sharma and coworkers (2013) on hydrolysis of cod liver oil using CRL for fatty acid production. The process was carried out at pH 7.0 [8]. Hydrolysis of soybean oil using CRL was also carried out at pH 7.0 [16]. In the research of Freitas and coworkers (2007) on hydrolysis of soybean oil by using 3 types of lipase, among them was CRL with pH for hydrolysis reaction being 7.0 [17].

### 3.4. Effect of Temperature

Temperature has a strong effect to hydrolysis degree of lipase. When increase temperature, lipase becomes flexible; thus the hydrolysis degree is also increased. Moreover, increasing temperature will decrease viscosity of mixture reaction. This makes lipase easy to bond to a substrate; therefore the hydrolysis degree is increased. However, very high temperature will denature lipase because lipase's nature is protein; as a result, hydrolysis degree is decreased [15].

As shown in Figure 1(d), the highest hydrolysis degree was obtained at 40°C. And this result was equivalent to some studies as hydrolysis of soybean oil by using CRL at 40°C of Freitas and coworkers (2007) [17]; Zhou and coworkers (2015) used CRL to hydrolyze Jatropha oil to produce biodiesel at 40°C [18]. Yiğitoğlu and Temoçin (2010) also conducted a research on hydrolysis of vegetables oil by using enzyme immobilized and free CRL at 40°C [19].

### 3.5. Hydrolysis Time

**Figure 2:**
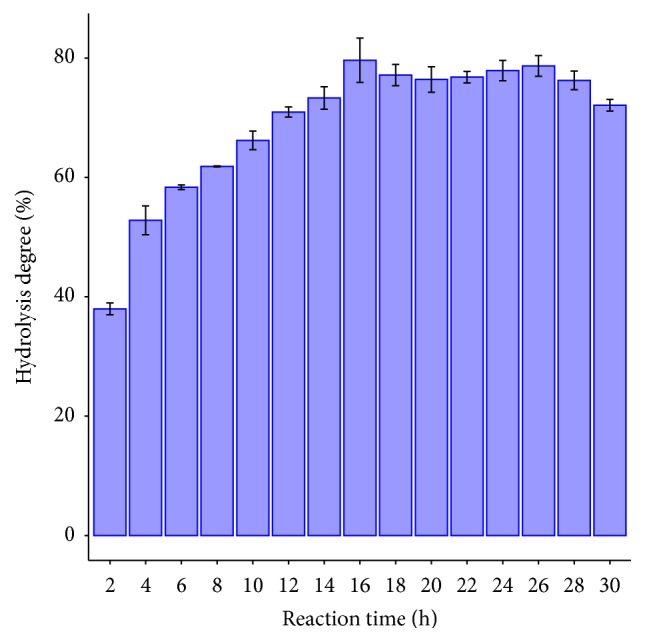
Time course of VCO hydrolysis catalyzed by CRL.

After 16 hours' reaction, the highest hydrolysis degree was achieved 79.64% (Figure 2). When comparing the result with other previous studies, hydrolysis degree of, for example, soybean oil by CRL was 70% after 24 hours' reaction [17]. On the other hand, tuna fish oil was hydrolyzed by using CRL, after 24 hours' reaction; the hydrolysis degree achieved was 86.5% [20]. In this research, the hydrolysis time was shorter than other studies, but it got equivalent hydrolysis degree. Hence, it can be understood that using iso-octane in this hydrolysis reaction had a positive effect. The presence of iso-octane in reaction mixture decreased VCO viscosity. VCO was emulsified easily and increased the interface of VCO and water, so this made CRL have more opportunity to contact with substrate and easily catalyze hydrolysis reaction [21]. As such, it shortened time course of VCO hydrolysis reaction.

### 3.6. Antibacterial Test

**Figure 3:**
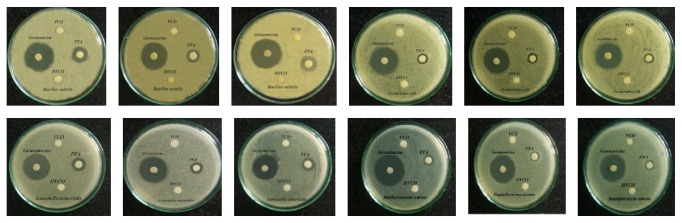
Antibacterial activity of VCO, HVCO, FFAs, and gentamicin against 4 types of tested bacteria.

According to Table 1 and Figure 3, FFAs showed clearly antibacterial activity on 4 types of bacteria. Diameter of inhibition zone of FFAs on each bacteria was different. FFAs' antibacterial activity on* Salmonella enteritidis* was stronger than* Staphylococcus aureus* and* Escherichia coli*, so the result demonstrated that FFAs extracted from hydrolyzed product were antibacterial agents. It was equivalent to some authors using FFAs in the form of pure chemicals. Shilling and coworkers (2013) showed the result of antibacterial ability of MCFAs (lauric acid in the form of pure chemicals) against* Clostridium difficile*. Moreover, hydrolyzed VCO also had an effect on the growth of* Clostridium difficile* because FFAs were released after hydrolysis of VCO [7]. Bergsson and coworkers (2002) studied antibacterial effect of fatty acids against* Helicobacter pylori* and the result exhibited that, among fatty acids, MCFAs were antibacterial agent [22]. The study of Kim and Rhee (2016) also showed that MCFAs could inhibit the growth of* Escherichia coli* [6].

VCO did not show its antibacterial activity against these tested bacteria. This was equivalent to the result of Shilling and coworkers (2013) in using VCO to inhibit* Clostridium difficile*, and VCO had a negative result [7].

HVCO in this study did not show its antibacterial ability although it might contain monoglyceride. This could be explained that, in this study, HVCO only contained a small amount of monoglyceride, so it did not have much enough to inhibit bacteria.

### 3.7. Minimum Inhibitory Concentration (MIC)

**Figure 4:**
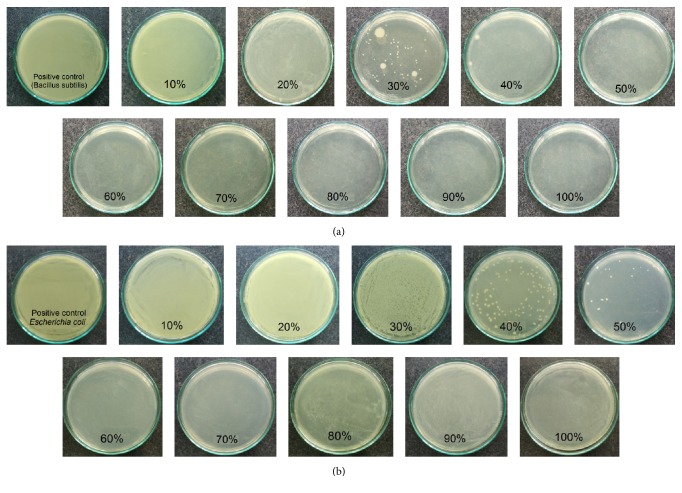
MIC of FFAs against* Bacillus subtilis* (a) and* Escherichia coli* (b).

**Figure 5:**
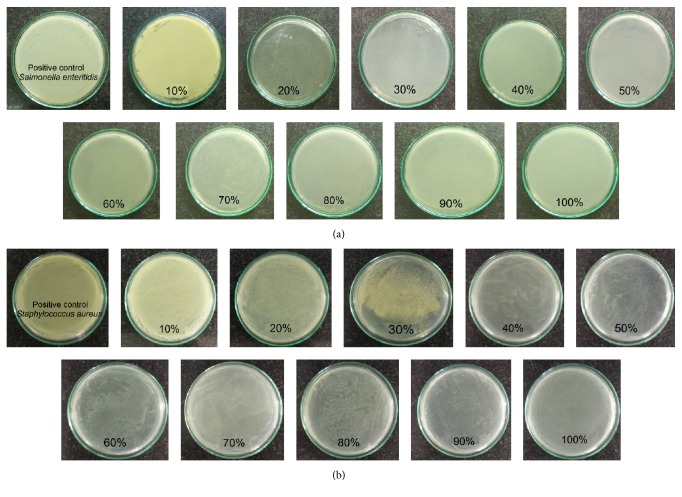
MIC of FFAs against* Salmonella enteritidis* (a) and* Staphylococcus aureus* (b).

The result of MIC was shown in Table 2 and Figures 4 and 5. The MIC of FFAs against* Salmonella enteritidis* was lowest while* Escherichia coli* needed concentration of FFAs up to 60% to inhibit it, so this showed that minimum concentration of FFAs to inhibit bacteria was completely different; it did not distinguish which is Gram-negative or Gram-positive bacteria.

## 4. Conclusions

This research found the best hydrolysis conditions of VCO under four parameters: VCO to buffer ratio, lipase concentration, pH, and temperature. Using lipase to hydrolyze VCO received the less changed FFAs because the hydrolysis reaction is conducted under milder condition of pH and temperature than acidic or alkaline hydrolysis. And enzymatic hydrolysis can avoid some danger from undesirable side-reaction, not affecting color of products or limiting oxidized products so that FFAs can be applied in other fields as food preservation, pharmaceuticals, and so forth. Moreover, using iso-octane to dissolve VCO saved the time of hydrolysis reaction compared to other researches. FFAs were obtained, isolated, and showed their antibacterial activity against Gram-positive and Gram-negative bacteria while VCO and HVCO did not show any positive results. MIC of FFAs was determined at different concentrations. Based on that, FFAs can be used as a preservative in food products with appropriate amount in our next researches.

## Figures and Tables

**Table 1:** Diameter of inhibition zone on 4 types of bacteria.

Bacteria	Diameter of inhibition zone ± SD (mm)		
	FFAs	HVCO	VCO
*Bacillus subtilis*	11.33 ± 1.15	6	6
*Escherichia coli*	8.67 ± 0.58	6	6
*Salmonella enteritidis*	10.33 ± 0.58	6	6
*Staphylococcus aureus*	8.33 ± 0.58	6	6

**Table 2:** Minimum inhibitory concentration of FFAs against four types of bacteria.

Bacteria	MIC (%)
*Bacillus subtilis*	50
*Escherichia coli*	60
*Salmonella enteritidis*	20
*Staphylococcus aureus*	40
